# MOF-Mediated Construction of NiCoMn-LDH Nanoflakes Assembled Co(OH)F Nanorods for Improved Supercapacitive Performance

**DOI:** 10.3390/nano14070573

**Published:** 2024-03-26

**Authors:** Zhou Wang, Yijie Lian, Xinde Zhu, Qi Wang

**Affiliations:** Key Laboratory of Liquid-Solid Structural Evolution and Processing of Materials of Ministry of Education, School of Materials Science and Engineering, Shandong University, Jinan 250061, China; wangzhou@sdu.edu.cn (Z.W.);

**Keywords:** layered double hydroxide, metal-organic framework, supercapacitor, core–shell structure

## Abstract

The application of transition metal hydroxides has long been plagued by its poor conductivity and stability as well as severe aggregation tendency. In this paper, a novel hierarchical core–shell architecture, using an F-doped Co(OH)_2_ nanorod array (Co(OH)F) as the core and Mn/Ni co-doped Co(OH)_2_ nanosheets (NiCoMn-LDH) as the shell, was constructed via an MOF-mediated in situ generation method. The obtained Co(OH)F@ NiCoMn-LDH composites exhibited excellent supercapacitive performance with large specific capacitance as well as improved rate capability and long-term stability. The effect of the Ni/Mn ratio on the supercapacitive performance and energy storage kinetics was systematically investigated and the related mechanism was revealed.

## 1. Introduction

Currently, the global energy structure has been reformed by renewable energy sources to cope with the increasing demands and environmental pollution issues of traditional fuels [1,2]. However, the application of renewable energy sources has long been plagued by its instability, seasonality, regionality, low conversion efficiency, etc. [3,4,5,6]. As a solution, energy storage devices are being used as buffer capacities in every level of the power grid, such as generation, transmission, and distribution [7,8]. Among various energy storage devices, the supercapacitor has received widespread attention due to its excellent power density, high charge–discharge rate, and long cycle stability [9,10]. However, compared to alkali-metal (Li, Na, K, Zn-ion) batteries, its lower energy density has long been a difficult hotspot in its application, which has broadly stimulated research interest in developing new electrode materials for supercapacitors that combine high energy density and high power density [11].

As a typical pseudocapacitive material, transition metal hydroxides (TMHs) have become a popular electrode material in pursuing high supercapacitive performance due to their high redox activity, adjustable composition, low cost, and environmental friendliness [12]. However, TMHs have poor conductivity and stability as well as a severe aggregation tendency [13,14,15], resulting in a more unsatisfying capacitive performance than expected. In recent years, many efforts such as morphological engineering, elemental doping, heterojunction assembly, and similar approaches have been made in realizing the potential of TMHs [16,17,18]. Among these strategies, metal/nonmetallic doping has attracted special attention, since it improves conductivity and produces extra active sites, as well as enhancing stability [19,20]. Fluorine has been applied as the nonmetallic doping element in many reports [21,22,23]. Due to the strong electronegativity of fluorine, the metal-fluorine (M-F) bond, with its deep ionicity and high polarization, is able to gather additional positive charges on the metal center for an efficient shuttling of the redox couple, which benefits the energy storage reaction. Moreover, F-doping is reported to be beneficial for reducing the thermodynamic and kinetic barrier [24], hence improving the conductivity and charge mobility as well as optimizing the adsorption energy of reactive species. Compared with nonmetallic doping, the metallic doping of TMHs, commonly known as layered double hydroxides (LDHs), has been systematically investigated [25,26,27]. The most obvious advantages of LDHs are their diverse compositions and metal–anion combinations, which offer substantial potential strength for composition design and leads to diverse function. Apart from that, the injected metallic ions are able to expand the interlayer space of LDHs and reduce mechanical stress during the charging and discharging processes, hence providing more active reaction sites and improving crystal structure stability.

LDHs, conventionally with nanosheet morphology, are prone to stack along the c-axis, resulting in decaying performance. Although the doping strategies are beneficial to the energy storage performance of LDHs in many aspects, things get tough when they are applied to deal with aggregation issues, which are a universal problem for materials with a layered structure. In that case, the strategy of anchoring LDH nanosheets on stable templates has been developed and proved efficient in many reports [28,29,30]. In this strategy, core–shell structures, applying template substances as the core and LDH as the shell, are normally constructed. The LDH-based core−shell architectures have been widely proven to be conducive to achieving improved energy storage performance [31,32,33]. Firstly, the anchoring effect decreases the stacking probability of LDH nanosheets with free movement, thus increasing the exposed active sites and improving stability. Moreover, the well-designed compositions can benefit from the unique properties (band structure, morphology, conductivity, etc.) of the substrate materials and realize a synergistic effect [34,35]. Given the poor conductivity of LDHs, materials with low resistance, such as carbon nanotubes [28], graphene [30], and carbon fibers [36], are widely chosen as the core to facilitate the electron transfer. However, the lower capacity of the carbon species should be noticed, which probably affects the specific capacitance. To ensure a high energy density and compact interface contact, two Co(OH)_2_-based substances with identical crystal structure but diverse ion doping (F doping and metallic doping) have been used for the core−shell design.

Herein, we report on a hierarchical structure based on core–shell architecture using a fluorine-doped Co(OH)_2_ nanorod array (Co(OH)F) as the core, which is enclosed by the Mn/Ni co-doped Co(OH)_2_ nanosheets (NiCoMn-LDH) shell. The NiCoMn-LDHs shell was in situ assembled on the Co(OH)F core through a two-step process mediated by metal-organic frameworks (MOFs). The Co(OH)F@NiCoMn-LDH composite combined the merits of metallic and nonmetallic doping, as well as the anchoring effect between the matrix and second phase. The effect of the Ni/Mn ratio on the structure and supercapacitive performance of Co(OH)F@NiCoMn-LDH was systemically investigated and the relative mechanism was discussed. The electrochemical tests demonstrate that Ni is beneficial for capacity and Mn improves the conductivity and ion transportation, while their synergetic effect facilitates the charge transfer at the interface. In addition, the Co(OH)F@NiCoMn-LDH with the optimum Ni/Mn molar ratio of 4:3 was assembled into an asymmetric supercapacitor, which obtained promising performance and should have the potential to make contributions in other electrochemical areas.

## 2. Materials and Methods

### 2.1. Reagents

All reagents were purchased from suppliers and were used directly without purification. Cobalt nitrate hexahydrate (Co(NO_3_)_2_·6H_2_O, 99%, AR), dimethylimidazole (C_4_H_6_N_2_, 98%), manganese chloride tetrahydrate (MnCl_2_·4H_2_O, 99%, AR), and N-methylpyrrolidone (NMP, C_5_H_9_NO, 99.5%) were purchased from Aladdin Biochemical Technology Co., Ltd., Shanghai, China. Hydrochloric acid (HCl, 37%), urea (CO(NH_2_)_2_, 99%, AR), anhydrous ethanol (C_2_H_6_O, ≥95%, AR), ammonium fluoride (NH_4_F, 96%, AR), nickel nitrate hexahydrate (Ni(NO_3_)_2_·6H_2_O, 98%, AR), nitric acid (HNO_3_, AR), and carbon black were purchased from Sinopharm Chemical Reagent Co., Ltd., Shanghai, China. Polyvinylidene fluoride (PVDF) was purchased from Yingze LZY Battery Sales Department (Taiyuan, China). Nickle foam was purchased from Tianyu Technology Development Co., Ltd., Heze, China. Active carbon (AC, AR) was purchased from Kuraray Co., Ltd., Tokyo, Japan.

### 2.2. Preparation of Co(OH)F Nanoneedles Array

Firstly, the purchased Ni foam was cut into small pieces with a size of 1 cm × 2 cm. The obtained pieces were ultrasonically cleaned with acetone and deionized water for 20 min to get rid of grease and dust, followed by immersion into 2 M HCl for 20 min so as to remove surface oxide impurities. The Ni foam pieces were rinsed with deionized water until the wash solution was neutral and then dried at 40 °C.

The Co(OH)F nanoneedles array was grown on nickel (Ni) foam via a hydrothermal process. Typically, 5 mmol cobalt nitrate hexahydrate (Co(NO_3_)_2_·6H_2_O), 5 mmol urea (CO(NH_2_)_2_), and 2.5 mmol ammonium fluoride (NH_4_F) were dissolved in 50 mL deionized water. After stirring for 30 min to reach a uniform state, the solution and a piece of Ni foam was sealed into a 70 mL Teflon-lined autoclave and heated at 95 °C for 12 h. When cooled to room temperature, the autoclave was unsealed and the Ni foam loaded with active substance was taken out, cleaned to neutral with deionized water, and dried. Finally, the dark pink Ni foam was annealed in Ar atmosphere at 350 °C for 1 h with a ramp rate of 2 °C/min and the obtained sample was denoted as Co(OH)F.

### 2.3. In Situ Generation of Co-ZIF on Co(OH)F Nanoneedles Array

The Co-Zeolitic Imidazolate Framework (Co-ZIF) was decorated on the surface of Co(OH)F nanoneedles through an in situ reaction at the solid–solution interface. Typically, 1.5 g dimethylimidazole (C_4_H_6_N_2_) was dissolved into a mixed solution composed of 5 mL water and 5 mL anhydrous ethanol, followed by continuous stirring to obtain a clear solution. Subsequently, the Co(OH)F sample prepared in Section 2.2 was soaked in the solution and allowed to react at room temperature for 4 h. After being washed several times with deionized water and anhydrous ethanol, the sample was dried in an air-circulating oven for 12 h, which was denoted as Co(OH)F@ZIF.

### 2.4. In Situ Generation from Co-ZIF to NiCoMn-LDH on Co(OH)F Nanoneedles Array

The in situ generation from Co-ZIF to NiCoMn-LDH was realized through a hydrothermal process. In a typical fabrication, nickel nitrate hexahydrate (Ni(NO_3_)_2_·6H_2_O) and manganese chloride tetrahydrate (MnCl_2_·4H_2_O) was mixed with 20 mL anhydrous ethanol and stirred until a uniform solution was obtained. Then, the solution and Co(OH)F@ZIF sample prepared in Section 2.3 were transferred into a 50 mL Teflon-lined autoclave, which was subsequently maintained at 120 °C for 4 h. After cooling to room temperature, the samples were washed several times with deionized water and anhydrous ethanol. The obtained samples were labelled as Co(OH)F@Ni*_x_*CoMn*_y_*-LDH, where *x* and *y* represent the approximate molar ratio of Ni/Mn, as detailed in Table 1. In the following sections, Co(OH)F@NiCoMn-LDH with no subscripted number denotes Co(OH)F@Ni_4_CoMn_3_-LDH especially. By measuring the weight difference of bare Ni foam and the loaded sample, the mass loading of Co(OH)F@NiCoMn-LDH was calculated to be ~10.7 mg cm^−2^.

### 2.5. Assembly of Asymmetric Supercapacitor Devices

The asymmetric supercapacitor devices were assembled using Co(OH)F@NiCoMn-LDH as a positive electrode, active carbon (AC) as a negative electrode and 6 M KOH as an electrolyte. The AC electrode material was fabricated from commercially purchased AC through an additional acid corrosion and oxidation process. Specifically, the commercial AC was dispersed into 50 mL nitric acid (40 wt%) and the mixed solution was kept in the water bath of 60 °C for 3 h. After the solution cooled to room temperature, the dispersion was centrifuged and washed with deionized water several times. The collected powder was dried and applied as an active material of the negative electrode. In a conventional process, the pretreated AC, polyvinylidene fluoride (PVDF), and carbon black with a mass ratio of 8:1:1 were mixed through grinding them in an agate mortar. An appropriate amount of N-methylpyrrolidone (NMP) was slowly added into the mixture, which was ground continuously until a uniform paste was obtained. Then the prepared slurry was evenly coated with an area of 1 cm^2^ on clean Ni foam using a scraper. Finally, the AC negative electrode was obtained after drying the coated Ni foam at 60 °C for 24 h.

## 3. Results

### 3.1. Physicochemical Properties

**Figure 1:**
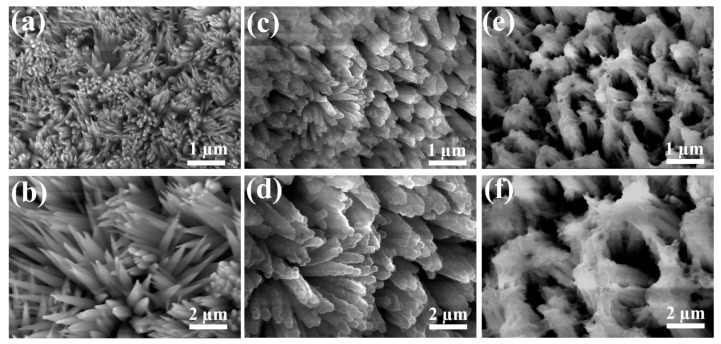
SEM images of (**a**,**b**) Co(OH)F, (**c**,**d**) Co(OH)F@ZIF, and (**e**,**f**) Co(OH)F@NiCoMn-LDH.

To verify the microstructure of the composites, the morphology of samples at each stage of the preparation process was characterized by scanning electron microscope (SEM). As exhibited in Figure 1a,b, uniform Co(OH)F nanoneedles were vertically grown on Ni foam. The nanoneedles are dense but show no adhesion with each other. Subsequently, when exposed to the C_4_H_6_N_2_ solution, Co^2+^ ions in Co(OH)F nanoneedles react with the organic ligands and generate Co-ZIF on the surface of nanoneedles. As a consequence, it can be seen from Figure 1c,d that ZIF particles shaped in polyhedron are strung along the nanoneedles. Finally, a solvothermal process destroys the dodecahedral granular ZIF structure, which is etched and converted into a thin sheet-like structure. The nanosheets uniformly covered and connected the neighboring nanoneedles, forming a porous interlinked structure (Figure 1e,f).

**Figure 2:**
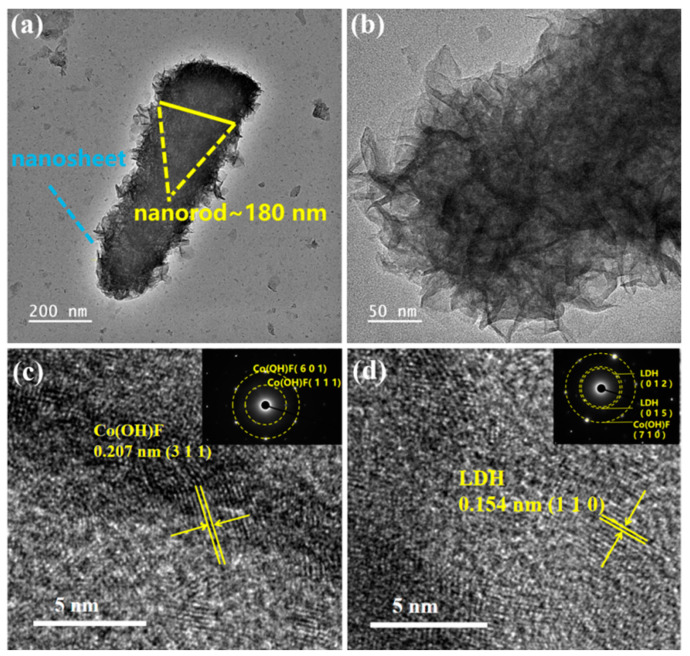
(**a**,**b**) TEM and (**c**,**d**) HRTEM images of Co(OH)F@NiCoMn-LDH. The insets in (**c**,**d**) show the SAED patterns of Co(OH)F and NiCoMn-LDH.

In order to investigate the microstructure, structure, and phase of the Co(OH)F@NiCoMn-LDH composite materials in detail, transmission electron microscope (TEM) tests were conducted in this study. As shown in Figure 2a, the composites consisted of thin layered LDH nanosheets wrapped around Co(OH)F nanoneedles, displaying a hierarchical core–shell structure. The diameter of the nanoneedle core was about 180 nm, and the surface was uniformly coated with a thin layer of the LDH shell. It can be clearly seen from Figure 2b that the LDH shell was self-assembled by rich and irregularly shaped nanosheets. On the one hand, the Co(OH)F nanoneedles provide a supporting framework for the layered structure, which can avoid LDH nanosheets stacking and facilitate electron transfer. On the other hand, the unique three-dimensional open network architecture, interconnected by LDH nanosheets, effectively increases the specific surface area and provides abundant active sites. Moreover, the multi-channel network structure provides better ion transport, which is conducive to ion infiltration, thus accelerating the redox process on the surface of electrode.

High-resolution TEM (HRTEM) images were recorded to investigate the lattice fringes, which helps to determine the phase of the composites. Figure 2c shows an interplanar spacing of 0.207 nm, corresponding to the (311) crystallographic plane of Co(OH)F. In Figure 2d, a narrower interplanar spacing of 0.154 nm is detected, which originates from the (110) plane of the LDH. Furthermore, the selected area electron diffraction (SAED) patterns of two different areas were captured, which both show scattered diffraction spots, testifying to their single-crystal structure. The pattern shown in the inset of Figure 2c can be calibrated into two sets of diffraction spots with lattice spacings of 1.504 nm and 2.522 nm, which can be ascribed to the (601) and (111) planes of Co(OH)F. Meanwhile, the pattern in the inset of Figure 2d is calibrated to be attributed to the (012), (015), and (710) planes of the LDH. The element distribution in Co(OH)F@NiCoMn-LDH was also characterized by energy dispersive spectroscopy (EDS) mapping. As displayed in Figure S1, Ni and Mn elements were uniformly located on the nanoneedle, indicating that a considerable amount of Co elements are evenly replaced by Ni and Mn, most probably forming NiCoMn-LDH. Figure S2 displays the content of each element, which confirms the injection of F with a considerable atomic concentration of 2.84%.

**Figure 3:**
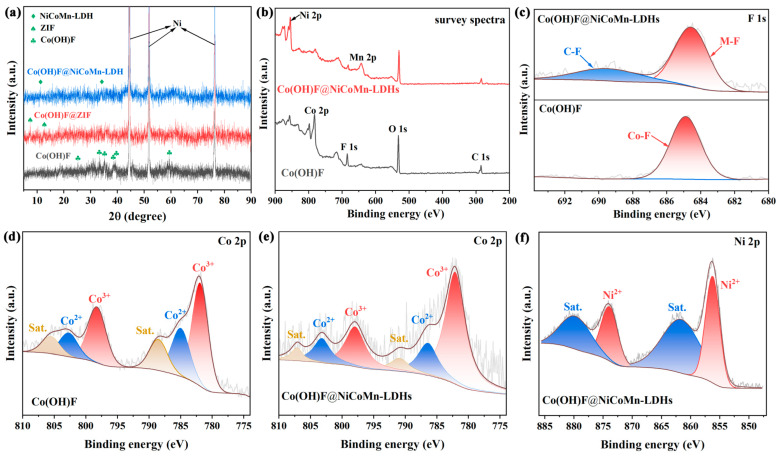
(**a**) XRD patterns of samples at different fabrication stages. (**b**) Survey XPS spectra and (**c**) F 1s spectra of Co(OH)F and Co(OH)F@NiCoMn-LDH. (**d**) Co 2p spectrum of Co(OH)F. (**e**) Co 2p and (**f**) Ni 2p spectra of Co(OH)F@NiCoMn-LDH.

Since the target composite material (Co(OH)F@NiCoMn-LDH) of this work was designed based on theory related to element doping, the crystal structure and the electronic structure need to be clarified first and foremost. Therefore, the X-ray diffraction (XRD) patterns of samples obtained at different fabrication stages were recorded (Figure 3a). The XRD pattern of Co(OH)F shows characteristic peaks at 25.7°, 33.5°, 35.7°, 38. 8°, 39.9°, and 59.1° (labeled with ♣), corresponding to the (210), (201), (111), (211), (410), and (002) crystal planes of Co (OH) F standard card (PDF#50-0827). After being treated with a C_4_H_6_N_2_ solution, Co(OH)F@ZIF exhibited two new peaks at low diffraction angles of around 7° and 16° (labeled with ♠), which is consistent with the result of ZIF in previous literature [37]. The diffraction peaks in the pattern of Co(OH)F@NiCoMn-LDH can barely be detected, which can be attributed to the disrupted long-range order of crystal lattice by the ultra-thin nanosheet morphology of NiCoMn-LDH. However, two weak peaks located at ~11.3° and ~34.4° (labeled with ♦) can still be distinguished, which fit the (003) and (012) planes of Ni(OH)_2_ standard card (PDF#38-0715), respectively. Considering the similar atomic radii of Ni, Co, and Mn, the above two new peaks are probably ascribed to the NiCoMn-LDH, which can be verified by element analysis.

The element composition and chemical valence state of Co(OH)F and Co(OH)F@NiCoMn-LDH was investigated by X-ray photoelectron spectroscopy (XPS). The survey spectra of both samples (Figure 3b) confirm the elementary composition of Co, F, and O for Co(OH)F, as well as the existence of Mn and Ni in Co(OH)F@NiCoMn-LDH, which is in agreement with the EDS-mapping result. In an effort to further analyze the electronic valence states of each element, the high-resolution XPS spectra were recorded. The O 1s spectra of both samples are divided into two overlapped peaks (Figure S3a). The peak at 531.7 eV is ascribed to metal hydroxide, while the one with lower intensity can be assigned to the surface hydroxyl groups [22]. Figure 3c shows the F 1s XPS spectra of Co(OH)F and Co(OH)F@NiCoMn-LDH, which can be fitted into one and two peaks, respectively. In both cases, the peak at around 684.7 eV is in close agreement with the F ion in metal hydroxyl fluoride [38]. In the case of Co(OH)F@NiCoMn-LDH, the weak additional peak with higher binding energy (BE) is due to the formation of C-F bonds during the in situ generation of ZIF [38].

The Co 2p spectra (Figure 3d,e) of both samples exhibit two contributions, 2p_1/2_ and 2p_3/2_, located at 798.3 eV and 782.1 eV. The coexistence of Co^2+^ and Co^3+^ is evidenced by a shoulder on the main peak at the low-BE direction. Upon deconvolution, the 2p_1/2_ and 2p_3/2_ peaks are, respectively, fitted into three signals. Peaks centered at 781.9 eV and 798.3 eV are attributed to Co^3+^, while those at 785.1 eV and 802.8 eV are ascribed to Co^2+^. The shakeup satellite peaks at higher BE originate from the 3d unpaired electrons of Co^2+^ [22]. By calculating the peak area of the respective ions, the ratio of Co^3+^/Co^2+^ was estimated, resulting in a value of 1.90 for Co(OH)F and 3.36 for Co(OH)F@NiCoMn-LDH. The higher valence state of Co in Co(OH)F@NiCoMn-LDH should be the result of oxidation during the second hydrothermal process.

The valence state of introduced elements (Ni and Mn) in Co(OH)F@NiCoMn-LDH was also investigated in detail. The Ni 2p spectrum (Figure 3f) was fitted by considering two resolved doublets of 2p_1/2_ and 2p_3/2_, which are, respectively, divided further into two signal peaks. As marked in the figure, peaks at 856.3 eV and 874.1eV are the characteristic signal of Ni^2+^, while the broad peaks located at the higher BE direction are the satellite peaks of Ni^2+^ [39]. The Mn 2p 2p_3/2_ spectrum (Figure S3b) shows a broad peak centered at 644.5 eV, which is fitted into two Gaussian peaks at 639.7 eV and 644.8 eV, corresponding to Mn^2+^ and Mn^3+^, respectively [40].

### 3.2. Optimization of Ni/Mn Ratio

**Figure 4:**
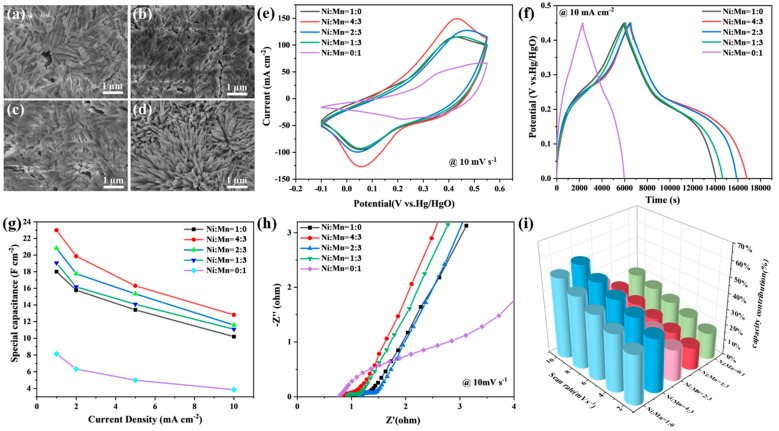
SEM images of Co(OH)F@Ni*_x_*CoMn*_y_*-LDH with different Ni/Mn ratios: (**a**) 1:0, (**b**) 2:3, (**c**) 1:3, and (**d**) 0:1. Electrochemical performance of Co(OH)F@Ni*_x_*CoMn*_y_*-LDH: (**e**) CV curves at 10 mV s^−1^, (**f**) GCD curves at 1 mA cm^−2^, (**g**) specific capacitances at different current densities, (**h**) Nyquist plots, and (**i**) capacitive-controlled contribution to the total pseudocapacitive charge storage.

In an effort to tap the potential of our design, the Ni/Mn ratio of the solution in the second hydrothermal reaction was optimized. As listed in Table 1, five groups of solution with different Ni/Mn ratios were applied. Firstly, the SEM images of all Co(OH)F@Ni*_x_*CoMn*_y_*-LDH samples were captured to investigate the effect of Ni/Mn ratio on morphology. As shown in Figure 1e,f and Figure 4a–d, the Ni/Mn ratio is critical to the generation of LDH nanosheets. All Co(OH)F@Ni*_x_*CoMn*_y_*-LDH samples showed a rough surface; however, only Co(OH)F@Ni_4_CoMn_3_-LDH showed an obvious core–shell architecture with nanosheets wrapped around the surface of nanoneedles. The SEM images of Co(OH)F@Ni*_x_*CoMn*_y_*-LDH samples (Figure 4a–c) with the Ni/Mn ratios of 1:0, 2:3, and 1:3 displayed identical morphology, in which nanorods with a rough surface adhered tightly to each other. In the case of Co(OH)F@Ni_0_CoMn_1_-LDH (Figure 4d), the separated nanoneedles array was maintained but with no interconnection between them, which is not good for expanding the surface area or for charge transfer.

The electrochemical performances of different Co(OH)F@Ni*_x_*CoMn*_y_*-LDH samples were also evaluated in a three-electrode system (working electrode: Co(OH)F@Ni*_x_*CoMn*_y_*-LDH; reference electrode: Hg/HgO; counter electrode: Pt; electrolyte: 6 M KOH) to obtain an optimum Ni/Mn ratio. As displayed in Figure S4, the CV curves of each Co(OH)F@Ni*_x_*CoMn*_y_*-LDH sample were recorded at various scan rates (2–10 mV s^−1^), and displayed an oblique-rectangle shape with obvious wide redox peaks, which can be interpreted as complex redox reactions of Co^2+^/Co^3+^, Ni^2+^/Ni^3+^, and Mn^2+^/Mn^3+^. The quasi-symmetric shape of CV curves demonstrates the reversible faradaic reactions, which are favorable to pseudo-capacitive energy storage. The CV curves obtained at 10 mV s^−1^ are integrated in Figure 4e for horizontal comparison, which clearly establishes the superior capacitance of Co(OH)F@Ni_2_CoMn_1_-LDH on the basis of its area within the CV curve being the largest. The galvanostatic charge/discharge (GCD) test was also conducted to investigate the supercapacitive behavior of different Co(OH)F@Ni*_x_*CoMn*_y_*-LDH samples. Figure S5 reveals the GCD curves obtained at various current densities (1–10 mA cm^−2^). The charge/discharge time decreases with the increasing current density. All GCD curves show a quasi-triangle shape but with obvious changes in slope at around 0.2 V, which forms an inclined platform in the middle of each curve, indicating the pseudo-capacitive behavior. Comparison between different samples was also conducted based on the GCD curves captured at the same current density of 1 mA cm^−2^, as depicted in Figure 4f. Co(OH)F@Ni_1_CoMn_0_-LDH with only Mn doping resulted in the lowest specific capacitance at each current density, which lagged far behind the other samples. According to the specific capacitance calculated at 1 mA cm^−2^ (Table 2), Co(OH)F@Ni_1_CoMn_0_-LDH only delivers a specific capacitance of 8.135 F cm^−2^. In comparison, Co(OH)F@Ni_0_CoMn_1_-LDH with only Ni doping resulted in a much higher specific capacitance of 18.00 F cm^−2^. Despite this, the effect of Ni/Mn co-doping still surpasses pure Ni doping on elevating specific capacitance. Moreover, it can be concluded in the three co-doped samples that the specific capacitance grows with the increasing Ni/Mn ratio, demonstrating the major role of Ni in improving the capacity and the importance of Ni/Mn synergism. The above results are in good accordance with the previous reports [41], which demonstrate that Ni incorporation can improve the specific capacity due to the high electrochemical activity. Co(OH)F@Ni_4_CoMn_3_-LDH shows the longest discharge time among all samples, which demonstrates its large capacitance (23.01 F cm^−2^ at 1 mA cm^−2^) and is in accordance with the result of the CV test. The specific capacitances of each sample at various current densities are estimated based on the GCD curves in Figure S5, which are exhibited in Figure 4g. Co(OH)F@Ni_4_CoMn_3_-LDH not only maintains its leading position in terms of specific capacitance at each current density, but also shows superior rate capability with the highest capacitance retention of 0.56 (capacitance at 10 mA cm^−2^/capacitance at 1 mA cm^−2^).

In order to figure out the mechanism of the superior performance of Co(OH)F@Ni_4_CoMn_3_-LDH, the impedance test was conducted to investigate the electron transfer behavior. The calibrated Nyquist plots of all samples show an identical shape composed of a semi-circle in the high-frequency region and an oblique line in the low-frequency area (Figure 4h). The intercept in the high-frequency region represents the internal resistance (R_s_), which mainly consists of the intrinsic resistance of the electrode and electrolyte, as well as the contact resistance at the interface. Meanwhile, the diameter of the semi-circle is associated with the charge transfer resistance (R_ct_) at the electrode/electrolyte interface. The value of R_s_ and R_ct_ for each Co(OH)F@Ni*_x_*CoMn*_y_*-LDH sample was determined by fitting the Nyquist plots in Figure 4h. As listed in Table 2, Co(OH)F@Ni_1_CoMn_0_-LDH with only Mn doping resulted in the smallest R_s_ (0.819 Ω) but the largest R_ct_ (0.276 Ω), which illustrates that Mn is beneficial to improving conductivity but the charge transfer at the Mn sites is tough. As a consequence, the lowest specific capacitance was obtained. However, the charge transfer problem can be resolved by the additional injection of Ni. With an optimum Ni/Mn ratio of 4:3, the lowest R_ct_ (0.082 Ω) was achieved, as well as a satisfying R_s_ value of 0.827 Ω. Moreover, the slope of the Nyquist plot in the low-frequency region is universally applied to evaluate Warburg impedance (Z_w_) caused by the ions’ diffusive resistance between the electrolyte and electrode. It should be noticed that Co(OH)F@Ni_1_CoMn_0_-LDH shows significantly lower Z_w_ than the other samples, which proves that Mn improves ion transportation. Therefore, with the well facilitated electronic and ionic kinetics, Co(OH)F@Ni_4_CoMn_3_-LDH resulted in the largest specific capacitance. The above results are consistent with the previous theoretical and experimental analysis [42,43], which testifies that Mn injection is able to reduce resistivity by increasing the density of states near the Fermi level, as well as facilitate the adsorption of OH^−^ and the desorption of the H proton.

Recently, studies have been widely focused on the contribution of different energy-storage mechanisms to supercapacitive performance. As is commonly recognized, intercalation contributes to the battery-type capacitance and is diffusion controlled, while the faradic reaction is a capacitive-controlled process. Based on the difference in the control step, the contribution of different mechanisms can be distinguished according to the following equation [44]:

(1) $$i=k_{1}v+k_{2}v^{1/2}$$

where *k*_1_*v* represents the capacitive-controlled current and *k*_2_*v*^1/2^ denotes the diffusion-controlled current. Based on Equation (1), all CV curves in Figure S4 were fitted (Figures S6–S10) and the capacitive-controlled contribution for each sample at various scan rates was calculated, as illustrated in Figure 4i. The capacitive-controlled contribution normally increases with the accelerating scan rate, so as our samples. By comparing the capacitive-controlled contribution of each sample at the same scan rate, Co(OH)F@Ni_4_CoMn_3_-LDH was found to possess the highest capacitive-controlled contribution (34% at 2 mV s^−1^ and 54% at 10 mV s^−1^) among all samples at each scan rate, indicating its superior kinetics. The high percentage of capacitive-controlled contribution endows Co(OH)F@Ni_4_CoMn_3_-LDH with better rate capability, which is in good agreement with the previous rate capability test (Figure S5 and Figure 4g). The cycling stability of Co(OH)F@Ni_4_CoMn_3_-LDH was also studied by repeatedly performing the charge–discharge processes at the current density of 10 mA cm^−2^. The specific capacitances of every 250 charge–discharge cycles were calculated and the retention is displayed in Figure S11, which shows a capacitance retention of 73.31% after 4500 cycles for Co(OH)F@Ni_4_CoMn_3_-LDH. It should be noticed that Co(OH)F@Ni_1_CoMn_0_-LDH shows the lowest retention of 57.89% after 4500 cycles, which is far lower than the Mn incorporated samples, indicating that Mn doping is beneficial to the cycling stability.

### 3.3. Asymmetric Device

**Figure 5:**
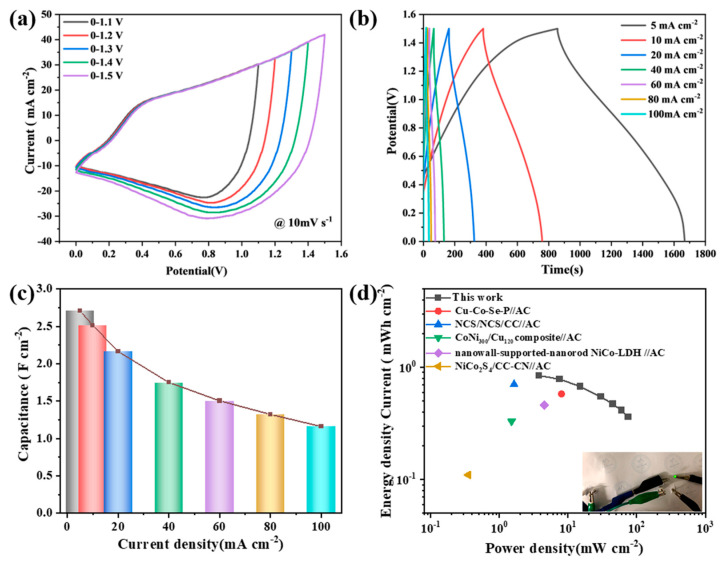
Electrochemical performance of Co(OH)F@NiCoMn-LDH//AC asymmetric device: (**a**) CV curves in different potential windows, (**b**) GCD curves, (**c**) specific capacitances at different current densities, (**d**) Ragone plots compared with similar asymmetric devices reported in the previous literatures. The inset in (**d**) shows the photograph of a green LED lit with two tandem asymmetric devices.

In order to further evaluate the potential of our material in practical applications, an asymmetric supercapacitor (ASC) device (denoted as Co(OH)F@NiCoMn-LDH//AC) was assembled by applying Co(OH)F@NiCoMn-LDH as the positive electrode and activated carbon (AC) as the negative electrode, respectively. The charge balance in ASC was achieved through matching the area of both electrodes according to their specific capacitance (Figure S12a), which was calculated from the GCD curves in Figures S5 and S12b, respectively. The working potential window of the ASC device is optimized by diagnosing the CV curves obtained in various voltage ranges. As shown in Figure 5a, the ASC device steadily expanded its working potential window up to 1.5 V with no sharp curling on the CV curve at the edge of the potential window.

The GCD curves of the ASC device were recorded at different current densities (5–100 mA cm^−2^). As shown in Figure 5b, all GCD curves exhibit a triangle-like shape, even the one recorded at a low current density of 5 mA cm^−2^. The specific capacitances at diverse current densities were calculated based on corresponding GCD curves and illustrated in Figure 5c. The ASC device demonstrated a high specific capacitance of 2.71 F cm^−2^, as well as a superior rate capability, which maintained 43.9% of the capacitance at a current density 19 times larger than the initial density.

Based on the data from the GCD curves in Figure 5b, the energy density (*E*, mWh cm^−2^) and power density (*P*, mW cm^−2^) of the Co(OH)F@NiCoMn-LDH//AC device were calculated according to the following formulas [45]:

(2) $$E=\frac{1}{7.2}CU^{2}$$

(3) $$P=\frac{E\;\times \;3600}{∆t}$$

where *C* (F cm^−2^), *U* (V), and Δ*t* (s) denote the specific capacitance, the potential window, and the discharge time, respectively. The results are shown as a Ragone plot in Figure 5d. Significantly, our Co(OH)F@NiCoMn-LDH//AC device delivers a remarkable power density of 75 mW cm^−2^ with an energy density of 0.363 mWh cm^−2^, as well as a high energy density of 0.845 mWh cm^−2^ at the power density of 3.75 mW cm^−2^. As summarized in Table S1 and Figure 5d, the superior capacitive performance of our device was demonstrated by comparing it to the recently reported asymmetric supercapacitive devices applying an AC electrode as the anode, such as Cu-Co-Se-P [46], NCS/NCS/CC [47], CoNi_300_/Cu_120_ composite [48], NiCo-LDH [49], and NiCo_2_S_4_/CC-CN [50].

## 4. Conclusions

In summary, this work successfully constructs a Co(OH)F@NiCoMn-LDH composite material, which consists of Co(OH)F nanoneedles wrapped with NiCoMn-LDH nanosheets. The Co(OH)F@NiCoMn-LDH electrode possesses an excellent specific capacitance of 23.01 F cm^−2^ at the current density of 1 mA cm^−2^, as well as a high rate capability and good cycling stability. Meanwhile, the asymmetric Co(OH)F@NiCoMn-LDH//AC device delivers a remarkable power density of 75 mW cm^−2^ at the energy density of 0.363 mWh cm^−2^. Furthermore, the effect of the Ni/Mn ratio on the morphology and supercapacitive performance is systematically investigated and the mechanism was revealed by electrochemical kinetics tests. This work helps us to better understand the synergistic effect of doping and morphology manipulation on energy storage behavior and provides a new way to construct high-performance materials with a hierarchical structure.

## Figures and Tables

**Table 1:** The added amount of raw materials for different Co(OH)F@Ni*_x_*CoMn*_y_*-LDH samples.

Sample Name	Ni(NO_3_)_2_·6H_2_O (g)	MnCl_2_·4H_2_O (g)	Ni/Mn (Molar Ratio)
Co(OH)F@Ni_1_CoMn_0_-LDH	1	0	1:0
Co(OH)F@Ni_4_CoMn_3_-LDH	0.66	0.33	4:3
Co(OH)F@Ni_2_CoMn_3_-LDH	0.50	0.50	2:3
Co(OH)F@Ni_1_CoMn_3_-LDH	0.33	0.66	1:3
Co(OH)F@Ni_0_CoMn_1_-LDH	0	1	0:1

**Table 2:** The specific capacitance, R_s_, and R_ct_ of Co(OH)F@Ni*_x_*CoMn*_y_*-LDH.

Ni/Mn	^1^ Specific Capacitance (F cm^−2^)	R_s_ (Ω)	R_ct_ (Ω)
1:0	18.00	1.077	0.214
4:3	23.01	0.827	0.082
2:3	20.82	1.180	0.127
1:3	19.06	0.942	0.102
0:1	8.135	0.819	0.276

^1^ The specific capacitance is calculated based on the GCD curves recoded at 1 mA cm^−2^.

## Data Availability

The data presented in this study are available on request from the corresponding author.

## Supplementary Materials

The following supporting information can be downloaded at: https://www.mdpi.com/article/10.3390/nano14070573/s1, Figure S1: EDS-mapping images of Co(OH)F@NiCoMn-LDH; Figure S2: The elemental composition of Co(OH)F@NiCoMn-LDH; Figure S3: (a) O 1s XPS spectra of Co(OH)F and Co(OH)F@NiCoMn-LDH, (b) Mn 2p_3/2_ XPS spectra of Co(OH)F@NiCoMn-LDH; Figure S4: CV curves of Co(OH)F@Ni*_x_*CoMn*_y_*-LDH with different Ni/Mn ratios; Figure S5: GCD curves of Co(OH)F@Ni*_x_*CoMn*_y_*-LDH with different Ni/Mn ratios; Figures S6–S10: Capacitive-controlled contribution to the total pseudocapacitive charge storage of Co(OH)F@Ni*_x_*CoMn*_y_*-LDH with different Ni/Mn ratios; Figure S11: Cycling stability of Co(OH)F@ Ni*_x_*CoMn*_y_*-LDH with different Ni/Mn ratios; Figure S12: (a) CV curves of AC and Co(OH)F@NiCoMn-LDH electrode, (b) GCD curves of AC electrode at different current densities; Table S1: Asymmetric supercapacitors devices and their energy density and power density reported in recent literature.
