# Supplementary material for: MOF-Mediated Construction of NiCoMn-LDH Nanoflakes Assembled Co(OH)F Nanorods for Improved Supercapacitive Performance
Source: Nanomaterials (Basel). 2024 Mar 26;14(7):573. doi: 10.3390/nano14070573 (PMC11013320; doi:10.3390/nano14070573)
Supplement: Supplementary file 1 [file nanomaterials-14-00573-s001.zip › nanomaterials-2902828-supplementary.pdf]

Supplementary Materials

MOF-mediated construction of NiCoMn-LDH nanoflakes assembled Co(OH)F nanorods for improved supercapacitive performance

Zhou Wang, Yijie Lian, Xinde Zhu* and Qi Wang*

Key Laboratory of Liquid-Solid Structural Evolution and Processing of Materials of Ministry of Education, School of Materials Science and Engineering, Shandong University, Jinan 250061, China; wangzhou@sdu.edu.cn (Z. W.)
*Correspondence: zhuxinde@sdu.edu.cn; wangqi1016@sdu.edu.cn

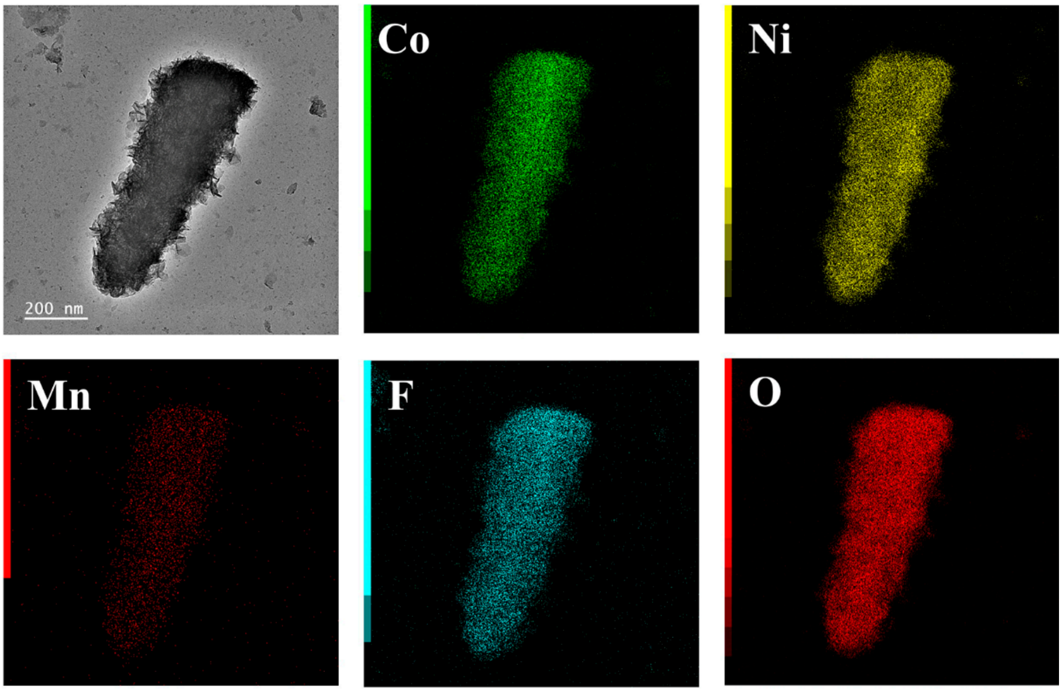

Figure S1. EDS-mapping images of Co(OH)F@NiCoMn-LDH.

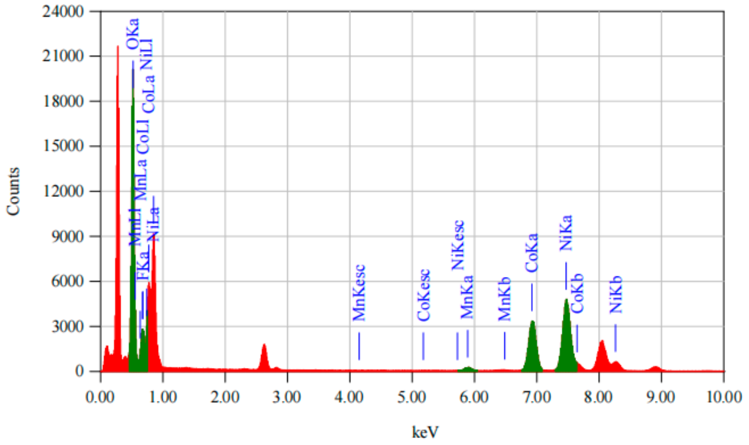

Acquisition Parameter
Instrument : JEM-F200(HRP)
Acc. Voltage : 200.0 kV
Probe Current: 7.47500 nA
PHA mode : T4
Real Time : 240.57 sec
Live Time : 214.95 sec
Dead Time : 9 %
Counting Rate: 3327 cps
Energy Range : 0 - 40 keV

Thin Film Standardless Standardless Quantitative Analysis
Fitting Coefficient : 0.4036

Element	(keV)	Mass%	Counts	Sigma	Atom%	Compound	Mass%	Cation	K
O K (Ref.)	0.525	38.11	111995.60	0.21	68.01				1.0000
F K	0.677	1.89	6306.27	0.05	2.84				0.8819
Mn K	5.894	1.07	3222.55	0.04	0.56				0.9801
Co K !	6.924	32.37	47960.71	0.32	15.68				1.9834
Ni K	7.471	26.55	67528.02	0.22	12.91				1.1551
Total		100.00			100.00				

Figure S2. The elemental composition of Co(OH)F@NiCoMn-LDH.

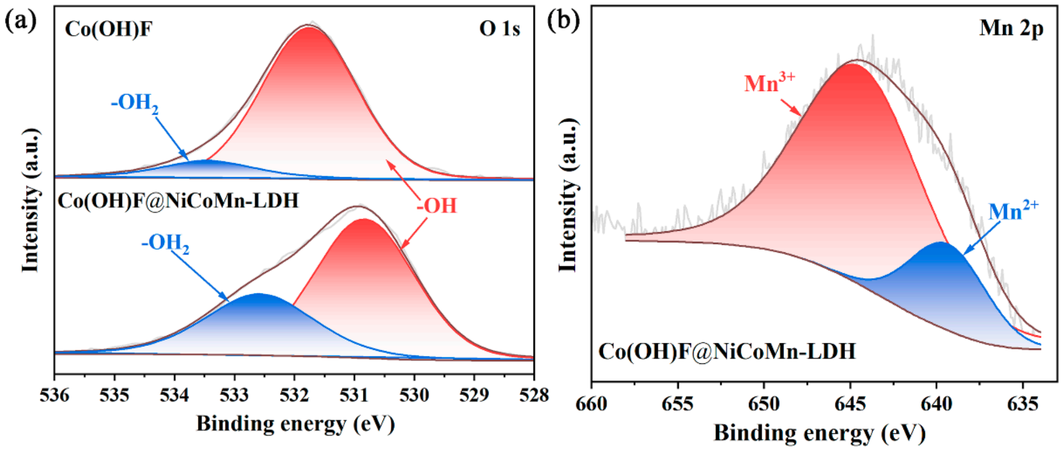

Figure S3. (a) O 1s XPS spectra of Co(OH)F and Co(OH)F@NiCoMn-LDH.
(b) Mn $2p_{3/2}$ XPS spectra of Co(OH)F@NiCoMn-LDH.

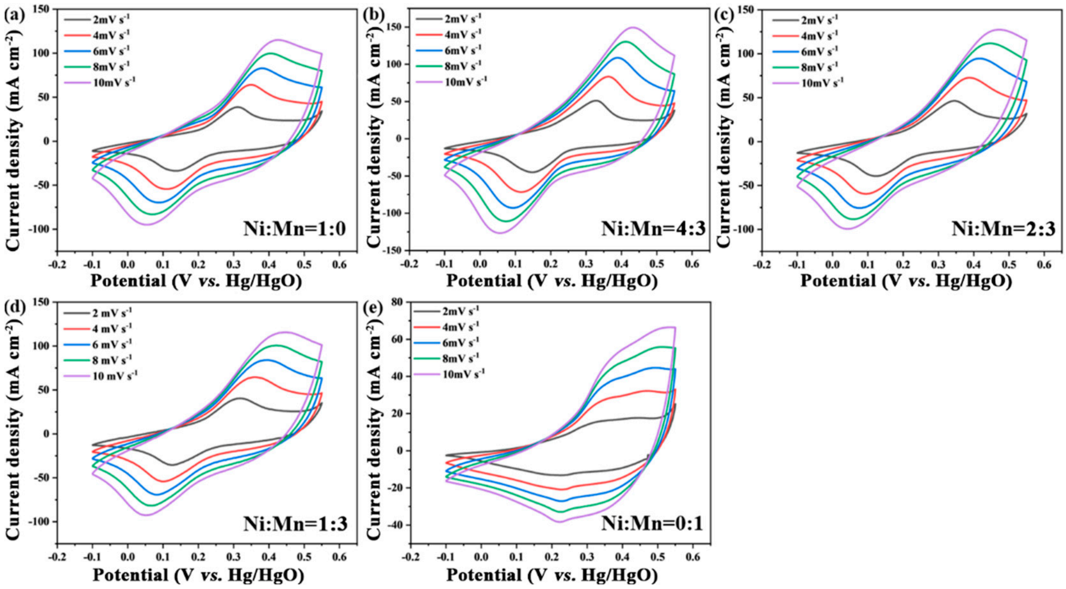

Figure S4. CV curves of Co(OH)F@Ni$_x$CoMn$_y$-LDH with different Ni/Mn ratios.

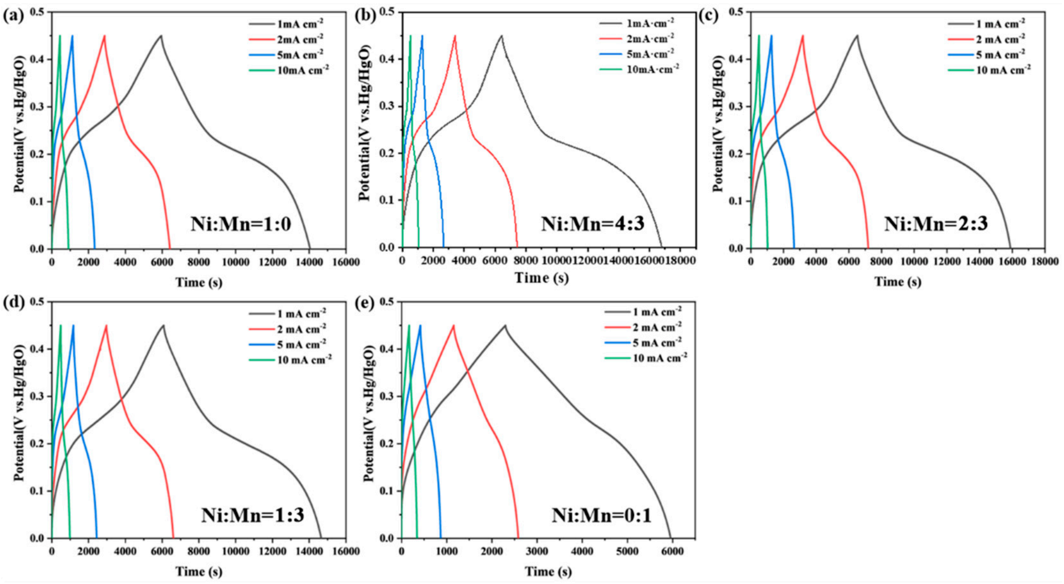

Figure S5. GCD curves of Co(OH)F@Ni_xCoMn_y-LDH with different Ni/Mn ratios.

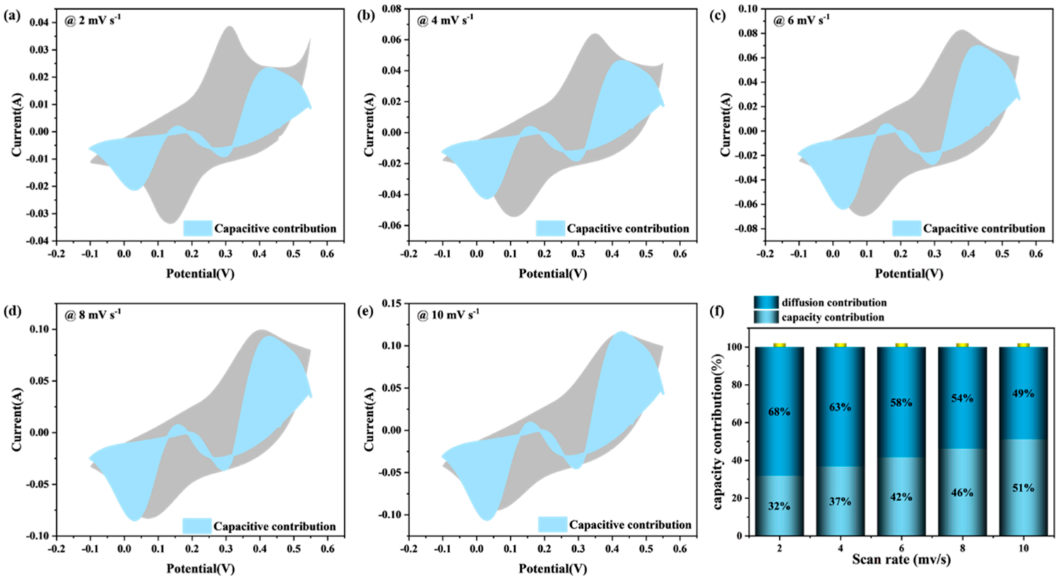

Figure S6. (a-e) CV curves and fitted capacitive-controlled curves of Co(OH)F@Ni_1CoMn_0-LDH at different scan rates. (f) Capacitive-controlled contribution to the total pseudocapacitive charge storage of Co(OH)F@Ni_1CoMn_0-LDH.

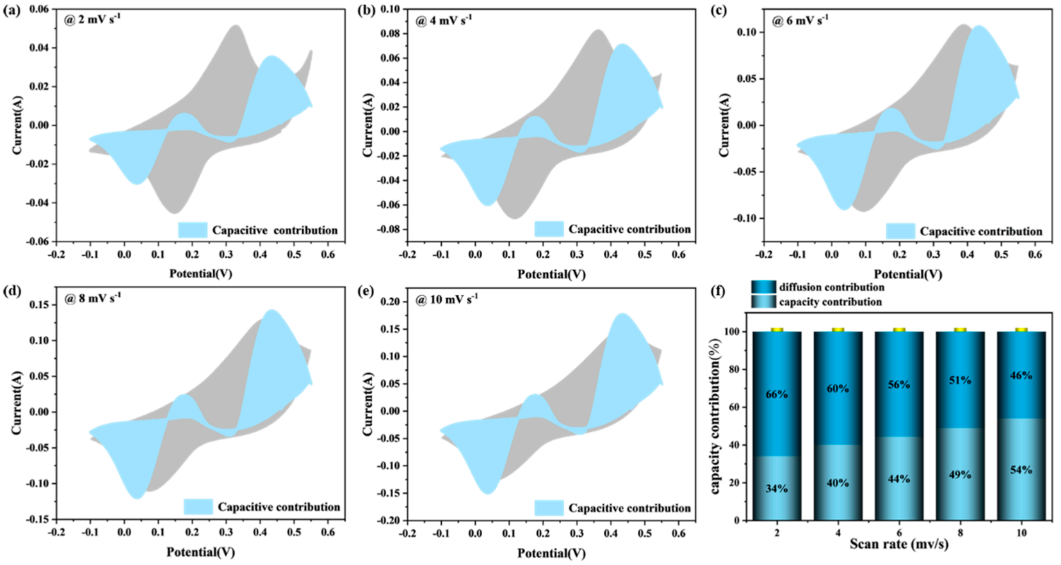

Figure S7. (a-e) CV curves and fitted capacitive-controlled curves of Co(OH)F@Ni_2CoMn_1-LDH at different scan rates. (f) Capacitive-controlled contribution to the total pseudocapacitive charge storage of Co(OH)F@Ni_4CoMn_3-LDH.

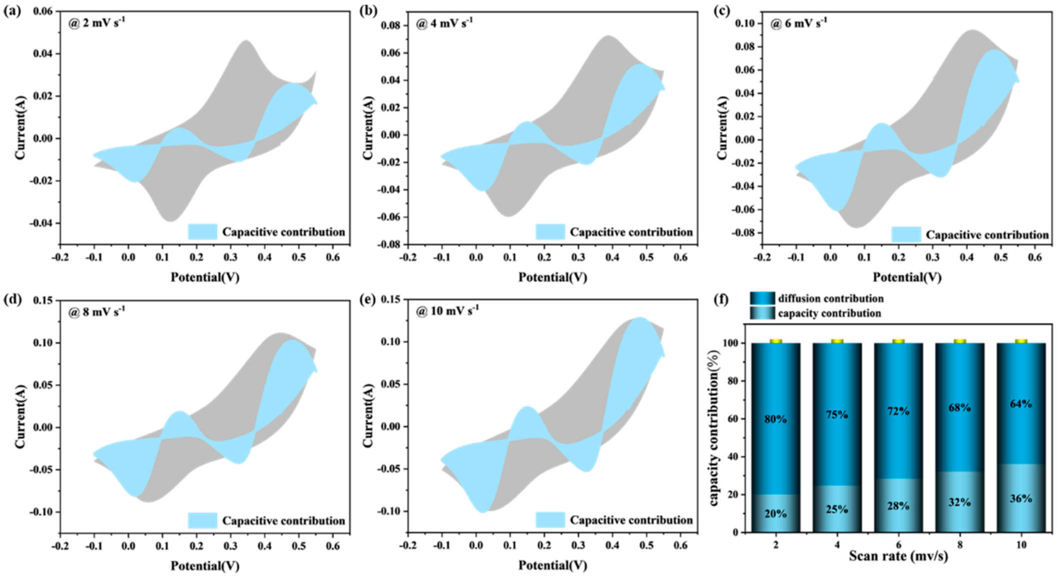

Figure S8. (a-e) CV curves and fitted capacitive-controlled curves of Co(OH)F@Ni_1CoMn_1-LDH at different scan rates. (f) Capacitive-controlled contribution to the total pseudocapacitive charge storage of Co(OH)F@Ni_2CoMn_3-LDH.

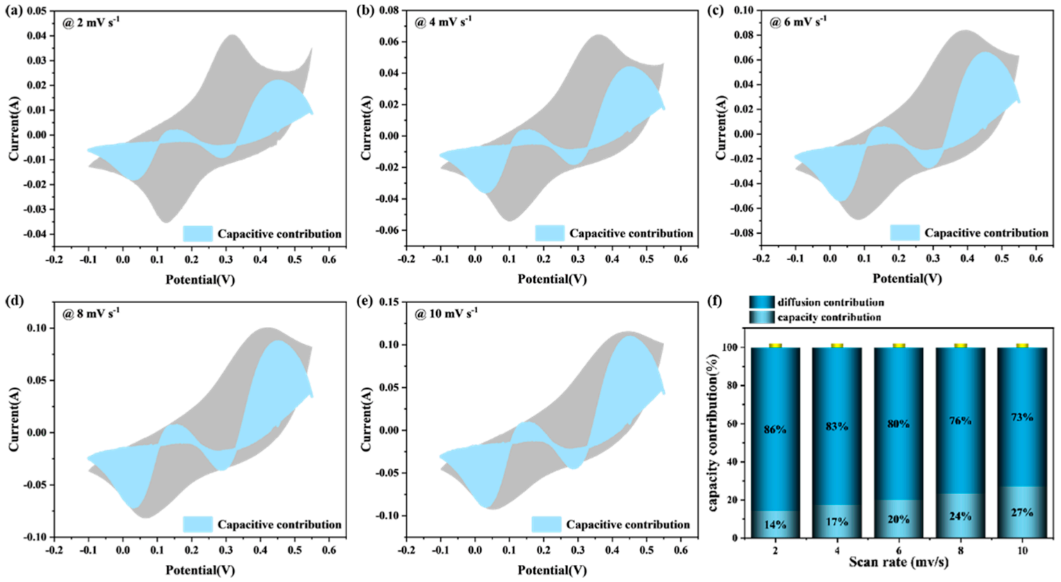

Figure S9. (a-e) CV curves and fitted capacitive-controlled curves of Co(OH)F@Ni_1CoMn_2-LDH at different scan rates. (f) Capacitive-controlled contribution to the total pseudocapacitive charge storage of Co(OH)F@Ni_1CoMn_3-LDH.

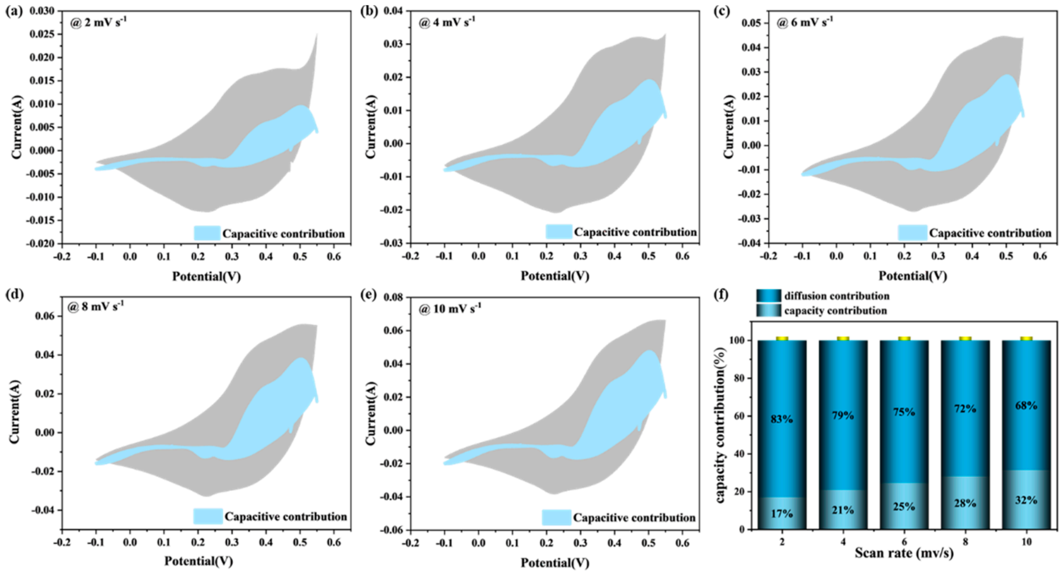

Figure S10. (a-e) CV curves and fitted capacitive-controlled curves of Co(OH)F@Ni_0CoMn_1-LDH at different scan rates. (f) Capacitive-controlled contribution to the total pseudocapacitive charge storage of Co(OH)F@Ni_0CoMn_1-LDH.

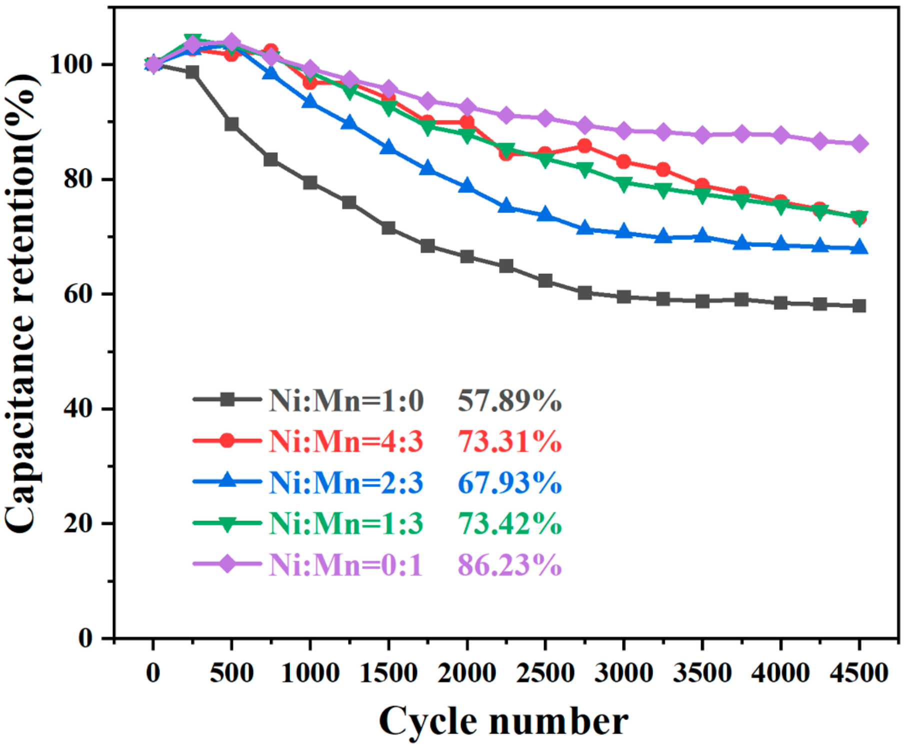

Figure S11. Cycling stability of Co(OH)F@Ni$_x$CoMn$_y$-LDH with different Ni/Mn ratios.

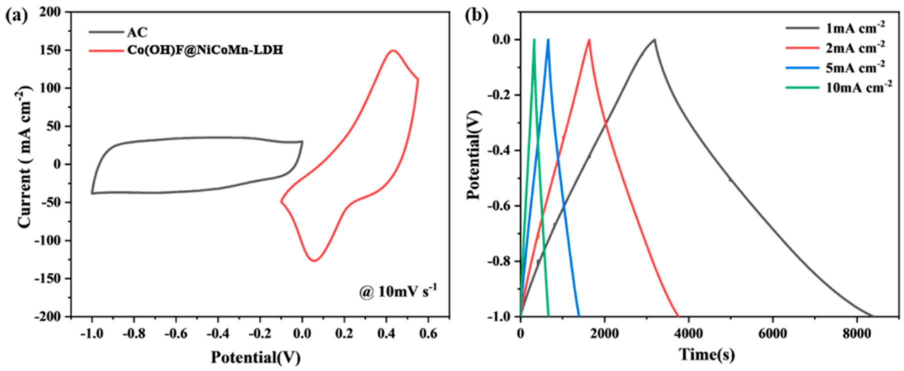

Figure S12. (a) CV curves of AC and Co(OH)F@NiCoMn-LDH electrode.
(b) GCD curves of AC electrode at different current densities.

Table S1. Asymmetric supercapacitors devices and their energy density and power density reported in recent literature

Active material of positive electrode	Power density (mW cm^{-2})	Energy density (mWh cm^{-2})	Ref.
Cu-Co-Se-P	8	0.58	[46]
NCS/NCS/CC	1.6303	0.7051	[47]
$CoNi_{300}/Cu_{120}$ composite	4.41	0.03086	[48]
NiCo-LDH	4.5	0.46	[49]
$NiCo_2S_4$/CC-CN	0.35	0.11	[50]
Co(OH)F@NiCoMn-LDH	75	0.363	This
Co(OH)F@NiCoMn-LDH	3.75	0.845	work
